# Notes and News

**Published:** 1898-08-06

**Authors:** 


### Notes and News.

(Collected and Communicated.)

The one convalescent home provided at Eastbourne for the sick soldiers of the British Army has about 65 patients passing through it yearly. This home is reserved for lung cases, and has no Army Sister attached.

The Society of Apothecaries has applied to the Charity Commissioners to be relieved of the trusteeship of the Physic Garden at Chelsea. The Trustees of the London Parochial Charities have offered to undertake the responsibility, and make provision for its maintenance for the purpose of botanical study.

A brass tablet and portrait have been placed inside the St. John Ambulance Station, St. Clement Danes, Strand, together with an inscription stating that the station has been erected as a memorial to the late Colonel Francis Duncan, C.B., M.P., director of the ambulance department of the association.

The sixth annual sermon of the Church Sanitary Association was preached on Sunday in the Westow Parish Church by the Rev. F. Lawrence, hon. sec., in the course of which he said Christ came to save the whole of man, body and soul alike. Therefore ought the ministers of the Church to aid sanitary authorities in securing for all fresh air, pure water, abundant light, proper food and drink, improved dwellings, wholesome surroundings, and the greatest possible immunity from infectious disease.

The War Office authorities have granted permission for the 300 boys of the Farm and Shaftesbury Schools, Bisley, to occupy the Eastbourne Redoubt from August 15th to 29th. It is the first time in the history of these country branches of the National Refuges for Homeless and Destitute Children that the boys have had the privilege of a fortnight at the seaside. Owing to the great expense in maintaining the 800 children in the society's eight homes and training ships "Arethusa" and "Chichester," the committee urgently solicit special help from the general public towards giving the Bisley boys their forthcoming treat without encroaching upon the funds needed for the other children. Contributions will be most gratefully acknowledged by the Secretary, 164, Shaftesbury Avenue, W.C.

A useful word of warning is given by Dr. Reifenacht Walters in regard to the somewhat extravagant suggestions which are now current as to the hygienic treatment of consumption. He says: "If at least two years are needed in Colorado or the Alps, a few months cannot be sufficient in England." There is no doubt that one of the limitations which we have to recognise to the extensive employment of open-air and hygienic treatment is that such methods require a long time. In the end, the good far outweighs the evil, including in this even the expense; still it has to be understood that it is nonsense to talk about cures in a couple of months.

Mrs. Millicent Garrett Fawcett writes to the *Times* in defence of Messrs. Bryant and May. She has investigated the charges made against them, she has interviewed the girls employed, she has made inquiries among C.O.S. workers and other experienced persons in Bow, she has had conversations and correspondence with Canon Wilberforce, and she says she has had "a long conversation with a man who, the Canon said, would 'open my eyes,'" and has made an analysis of papers entrusted to her by the same individual, and as a result she has come to the conclusion that nothing has been proved against the firm except the breach of the Factory Acts for which they have already been fined.

In answer to questions recently asked in the House of Commons by Mr. Channing and Mr. Steadman in regard to the case of a child, Frederick Edwards, whose death had been found by a coroner's inquest to have been caused by erysipelas set up by vaccination, Mr. Chaplin said the child in question was vaccinated, as stated, by the superintendent of the National Vaccine Establishment on May 24th. The lymph used was not glycerinated calf lymph, the child being vaccinated with twelve other children, at the same time and from the same calf, from arm to arm. The thirteen children were inspected seven days afterwards (on May 31st), and in all of them the vaccination had followed the normal course, and it was not until twenty days after vaccination that the child was brought again to the station and found to be suffering from erysipelas. Mr. Chaplin also pointed out that the ordinary period of incubation of erysipelas was from one to three days, and that the period which elapsed between vaccination and the appearance of the erysipelas showed that the latter was due not to the lymph, but to secondary infection during the third week after vaccination. Under the present law it is clear that in a case of this sort the vaccinator is held blameless. It will be interesting, however, to inquire what will be the position of affairs under the new Bill according to the provisions of which the public vaccinator will visit the house and there offer vaccination. How far will the vaccinator be responsible for the surroundings amid which he performs the operation? We take it that, even now, some sort of responsibility as to the child being in a fit state of health to bear the vaccination lies upon the operator. But when he visits will he not be responsible for the house as well, especially as it is proposed for him to have the option of declining to operate if the house appears hygienically defective? The matter is likely to be one of very considerable importance to public vaccinators.

